# Stress Concentration-Based Material Leakage Fault Online Diagnosis of Vacuum Pressure Vessels Based on Multiple FBG Monitoring Data

**DOI:** 10.3390/ma18204697

**Published:** 2025-10-13

**Authors:** Zhe Gong, Fu-Kang Shen, Yong-Hao Liu, Chang-Lin Yan, Jia Rui, Peng-Fei Cao, Hua-Ping Wang, Ping Xiang

**Affiliations:** 1 School of Civil Engineering and Mechanics, Lanzhou University, Lanzhou 730000, China; gz1304474184@163.com (Z.G.); shenfk19@lzu.edu.cn (F.-K.S.); liuyh2020@lzu.edu.cn (Y.-H.L.); 2 Lanzhou Vacuum Equipment Limited Liability Company, Lanzhou 730000, China; yancl_email@163.com; 3 Gansu Institute of Urban and Rural Planning and Design Institute Co., Ltd., Lanzhou 730000, China; richard800910@163.com; 4 School of Information Science and Engineering, Lanzhou University, Lanzhou 730000, China; caopf@lzu.edu.cn; 5 Key Laboratory of Mechanics on Disaster and Environment in Western China, Ministry of Education, Lanzhou University, Lanzhou 730000, China; 6 School of Civil Engineering, Central South University, Changsha 410075, China

**Keywords:** vacuum pressure vessel, leakage monitoring, quasi-distributed FBG sensors, stress concentration, vibration effect

## Abstract

Timely detection of leaks is essential for the safe and reliable operation of pressure vessels used in superconducting systems, aerospace, and medical equipment. To address the lack of efficient online leak detection methods for such vessels, this paper proposes a quasi-distributed fiber Bragg grating (FBG) sensing network combined with theoretical stress analysis to diagnose vessel conditions. We analyze the stress–strain distributions of vacuum vessels under varying pressures and examine stress concentration effects induced by small holes; these analyses guided the design and placement of quasi-distributed FBG sensors around the vacuum valve for online leakage monitoring. To improve measurement accuracy, we introduce a vibration correction algorithm that mitigates pump-induced vibration interference. Comparative tests under three leakage scenarios demonstrate that when leakage occurs during vacuum extraction, the proposed system can reliably detect the approximate leak location. The results indicate that combining an FBG sensing network with stress concentration analysis enables initial localization and assessment of leak severity, providing valuable support for the safe operation and rapid maintenance of vacuum pressure vessels.

## 1. Introduction

Pressure vessels are hermetically sealed containers designed to withstand internal or external pressures from gases or liquids, extensively used in aerospace, energy, superconducting systems, and medical devices for storage, separation, and heat exchange [1,2,3,4,5,6,7]. Cryogens, such as liquid helium or nitrogen, are used to sustain superconductivity [8]. For example, vacuum insulation in magnetic resonance imaging (MRI) superconducting magnets establishes a temperature barrier of nearly 286 K between ambient conditions (≈290 K) and the 4 K liquid helium chamber. The effectiveness of vacuum insulation depends directly on the internal vacuum level, which requires high-performance vacuum pumps to achieve very low internal pressure to maintain an adequate vacuum degree and thereby minimize heat transfer. Loss of vacuum insulation allows external heat ingress, accelerating cryogen boil-off and potentially causing a quench—a sudden loss of superconductivity—that can damage equipment. Reported failures in MRI systems due to inadequate helium management underscore the importance of reliable vacuum monitoring and maintenance. For instance, in the 1.5 T Avanto MRI system at Panyu Central Hospital in Guangzhou, a quench occurred following a series of events: a failure of the water chiller, delayed troubleshooting by the maintenance provider, and improper switching to municipal water cooling. This led to pipe rupture and water spray that short-circuited the quench protection circuit. This case highlights the critical importance of proper maintenance of vacuum insulation systems to ensure the stable operation of superconducting equipment.

In spacecraft systems, the sealing performance and reliability of pressure vessels are directly related to mission success. Harsh environments—including extreme thermal cycling, electromagnetic interference, mechanical vibrations, and micrometeoroid impacts—make space pressure vessels particularly vulnerable. Timely leakage monitoring can therefore extend spacecraft service life and enhance safety. Several notable incidents, such as a 1.5-mm breach on an ISS module in 2018 and a 0.8-mm puncture on Soyuz MS-22 in 2022, emphasized the necessity of real-time leak detection systems for pressure vessels [9,10].

Conventional leak detection methods—including pressure gauges, helium mass spectrometry, acoustic emission, ultrasonic testing, infrared thermography, visual inspection, and pressure decay techniques—each have their strengths and limitations [11,12]. For instance, helium mass spectrometry offers very high sensitivity, but is complex and costly for in-service systems [13]. Acoustic and ultrasonic methods can be influenced by wall thickness and material properties [14,15,16,17,18]. Infrared thermography and visual inspection rely heavily on surface and environmental conditions [19,20,21,22,23,24]. Pressure decay tests can detect leaks but cannot accurately localize them [25]. Acoustic resonance methods are limited to metal materials and media interference [26]. In general, the existing detection methods are difficult to configure a distributed sensor network on operational vessels for continuously and reliably monitoring the working state.

Optical fiber sensing technology, particularly fiber Bragg gratings (FBGs), offers compact size, high sensitivity, immunity to electromagnetic interference, corrosion resistance, and inherent electrical isolation [27,28,29,30,31,32,33,34], making it highly suitable for distributed structural monitoring. Prior studies have applied FBGs to monitor stress, strain, and damage in pressure vessels and related structures [35,36,37,38,39]. However, their use for online leak localization in metallic vacuum vessels remains less explored. For example, Degrieck et al. (2001) tested fiber-wound pressure vessels with embedded FBGs for online monitoring of composite structures [35]. Researchers at Harbin Institute of Technology (2006) mounted FBGs on circumferential and axial surfaces of FRP pressure vessels during pressurization [36]. Polish researchers (2007) employed FBGs to monitor local strains in high-pressure composite hydrogen vessels [37]. Scholars from Taiwan Province of China (2018) designed an FBG sensor system to monitor the pressure changes in an underwater pressure vessel at different dive depths and demonstrated that the FBG sensor system can provide accurate measurements of the structural strength of an underwater pressure vessel [38]. Norwegian scholars (2019) used embedded optical fibers to monitor damage due to impacts on fiber-wound composite pressure vessels [39]. In summary, existing research primarily focuses on stress/strain monitoring and damage detection in pressure vessel structures. It is hypothesized that co-designing the mechanical analysis of vacuum pressure vessels with a quasi-distributed optical fiber sensing network can provide a practical leakage monitoring system with advanced identification algorithms to detect and localize leak.

Given the analysis above, the stress–strain change in the cylindrical pressure vessel under different pressure conditions based on the Lame formulas has been characterized theoretically, and also the stress concentration coefficient of the hole edge under the condition of the cylinder with a hole subjected to the internal pressure is obtained for reflecting the leakage-induced mechanical state of vacuum pressure vessels. Quasi-distributed optical fiber sensors and sensor layout have thus been designed to construct a monitoring system for leakage fault identification and localization of vacuum pressure vessel, and data analysis under different leakage conditions has been carefully explored and characterized.

## 2. Structural Leakage Monitoring Mechanism of Pressure Vessels

### 2.1. Theoretical Description

**Figure 1:**
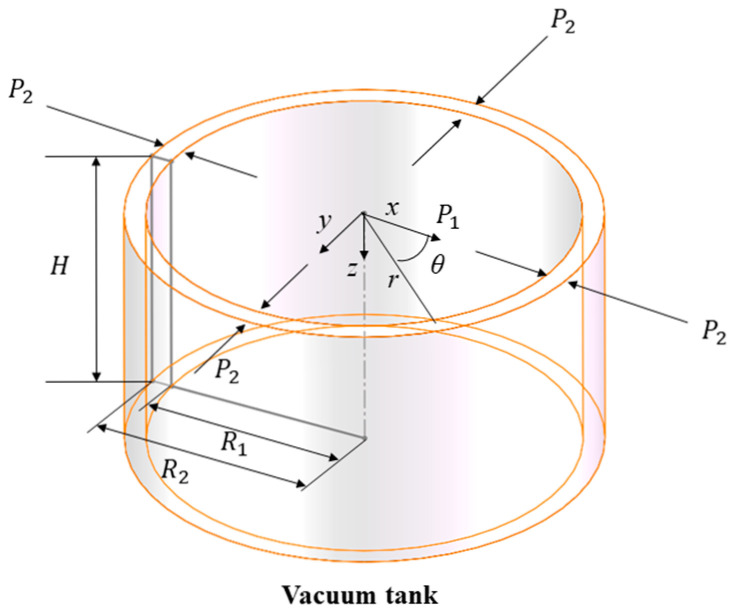
Refined model of the vacuum vessel.

For stainless steel vacuum tanks, both the vacuum extraction process and the vacuum unloading process can be equivalently modeled as a thick-walled cylindrical shell subjected to a constant external pressure equal to the standard atmospheric pressure and varying internal pressures. A cylindrical coordinate system is used with the axial direction defined as the *z*-axis. Figure 1 shows the inner radius $R_{1}$, outer radius $R_{2}$, cylinder height H, internal pressure $P_{1}$ and external pressure $P_{2}$, with atmospheric pressure denoted by $P_{0}$. Because radial stress cannot be neglected, the stress state is triaxial and shear stress components vanish. Stresses vary only with radius. Circumferential displacement is negligible, and only radial and axial displacements are considered.

The circumferential, radial, and axial stresses in a thick-walled cylinder under internal and external pressure are given by Lame’s formulas, as derived in Reference [40]:

(1) $$\sigma_{r}=\frac{P_{1}{R_{1}}^{2}-P_{2}{R_{2}}^{2}}{{R_{2}}^{2}-{R_{1}}^{2}}-\frac{(P_{1}-P_{2}){R_{1}}^{2}{R_{2}}^{2}}{{R_{2}}^{2}-{R_{1}}^{2}}\frac{1}{r^{2}}$$

(2) $$\sigma_{\theta }=\frac{P_{1}{R_{1}}^{2}-P_{2}{R_{2}}^{2}}{{R_{2}}^{2}-{R_{1}}^{2}}+\frac{(P_{1}-P_{2}){R_{1}}^{2}{R_{2}}^{2}}{{R_{2}}^{2}-{R_{1}}^{2}}\frac{1}{r^{2}}$$

(3) $$\sigma_{z}=\frac{P_{1}{R_{1}}^{2}-P_{2}{R_{2}}^{2}}{{R_{2}}^{2}-{R_{1}}^{2}}$$

**Figure 2:**
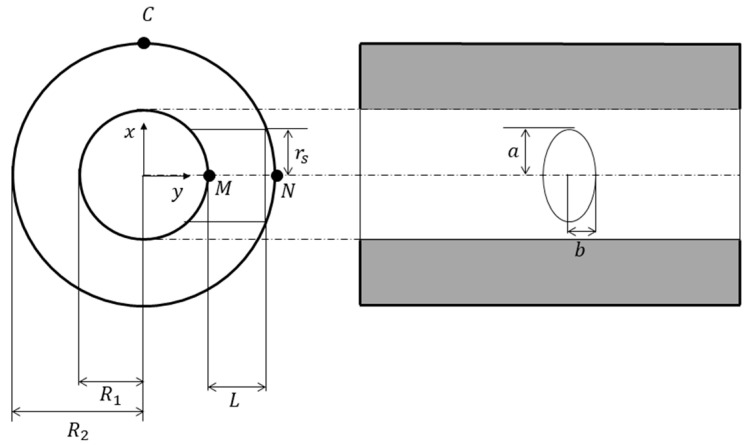
Geometric image of a cylinder with a hole.

**Figure 3:**
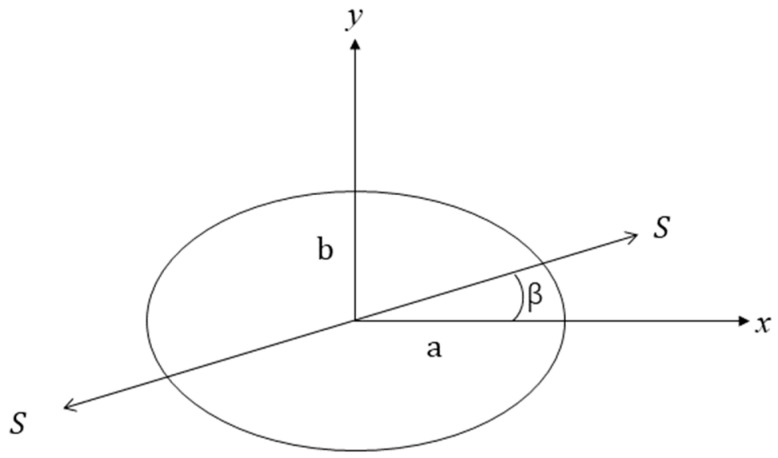
Elliptical hole subject to unit stress.

Reference [41] investigates the effect of introducing elliptical and circular lateral openings in a thick-walled cylinder under internal pressure. Figure 2 illustrates the perforated cylinder from two viewpoints, while Figure 3 shows an elliptical hole in an infinite elastic plate under tensile loading. For an infinite elastic plate containing a circular or elliptical hole under biaxial stress, the analytical solution obtained via conformal mapping is given as follows [41]:

(4) $$\begin{array}{l} \sigma_{x}+\sigma_{y}=\\ \frac{S\left[1-m^{2}-2\cos2\left(\beta -\nu \right)+2m\cos2\nu \cos2\left(\beta -\nu \right)-2m\sin2\nu \sin2\left(\beta -\nu \right)\right]}{1+m^{2}-2m\cos2\nu } \end{array}$$

where $\sigma_{x}$ and $\sigma_{y}$ denote the stresses along the x and y axes, respectively; S represents the unit stress; $m=\left(a-b\right)/\left(a+b\right)$, where a is the semi-major axis of the ellipse and b is the semi-minor axis; β is the angle between the loading direction and the major axis; and ν represents the angular position of any point on the ellipse’s circumference relative to the major axis.

When the loading direction aligns with the x axis (β = 0), the stresses at the endpoints of the elliptical axes are calculated. At the endpoints of the minor axis, ν = π/2, $\sigma_{y}$ = 0, according to Equation (4):

(5) $$\sigma_{x}=S_{x}\left[1+2b/a\right]$$

At the endpoints of the major axis, $\sigma_{x}$, β and ν are all zero, which gives

(6) $$\sigma_{y}=-S_{x}$$

When the load is applied along the y axis, the stresses at the endpoints of the minor axis can be obtained by setting $\beta =\nu =\frac{\pi }{2}$ and $\sigma_{y}=0$ in Equation (4):

(7) $$\sigma_{x}=-S_{y}$$

At the endpoints of the major axis, $\sigma_{x}=\nu =0$, $\beta =\frac{\pi }{2}$, it gives

(8) $$\sigma_{y}=S_{y}\left[1+2a/b\right]$$

Based on the above derivation, the stress state at any point along the elliptical circumference under arbitrary biaxial loading can be determined by substituting the appropriate values of $\sigma_{x}$, $\sigma_{y}$ and β into Equation (4). For a circular hole, the solution reduces to Equation (4) with a = b. Accordingly, the stress concentration factor for a plate with a circular hole under uniaxial loading is 3.

The subsequent analysis focuses on cylindrical vessels with lateral openings, specifically addressing stress concentrations induced by circular or elliptical side holes under internal pressure. For elliptical holes, the major axis is oriented perpendicular to the cylinder’s longitudinal axis.

The mechanical analysis in this study is conducted within the framework of plate and shell theory. The geometric complexity of the investigated structure arises from the presence of side and internal holes, with the stress field exhibiting multi-dimensional coupling under internal pressure loads. To achieve high-precision numerical analysis, the stress superposition principle is employed to simplify the problem: by introducing a hydrostatic stress state, the system is decomposed into a linear combination of the uniform response under basic pressure loading and local disturbance effects caused by the openings. This approach is theoretically justified because the hydrostatic stress component has negligible influence on the deformation mode, thereby preserving the primary mechanical behavior of the original system.

**Figure 4:**
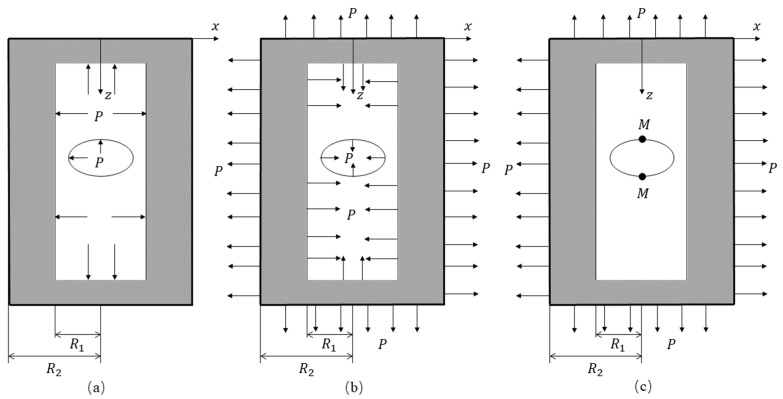
(**a**) The closed cylinder with side hole subjected to internal pressure; (**b**) the closed cylinder with side hole subjected to hydrostatic tension; (**c**) Case (**a**) + Case (**b**).

Figure 4a illustrates a cylindrical vessel with an elliptical lateral hole subjected to an internal pressure $P_{1}=P$. A hydrostatic tensile stress equal in magnitude to the internal pressure is then applied, as shown in Figure 4b. The combined loading condition resulting from this superposition is depicted in Figure 4c. Since the superimposed pressure is opposite to the direction of the internal pressure, the actual external pressure on the cylinder in Figure 4c is $P_{2}=-P$, and the internal pressure $P_{1}=0$. It can be precisely analyzed through Equations (1)–(3).

Therefore, for the thick-walled cylinder in Figure 4c, the three-dimensional stress can be obtained:

(9) $$\sigma_{r}=-\frac{P_{2}{R_{2}}^{2}}{{R_{2}}^{2}-{R_{1}}^{2}}\left(1-\frac{{R_{1}}^{2}}{r^{2}}\right)$$

(10) $$\sigma_{\theta }=-\frac{P_{2}{R_{2}}^{2}}{{R_{2}}^{2}-{R_{1}}^{2}}\left(1+\frac{{R_{1}}^{2}}{r^{2}}\right)$$

(11) $$\sigma_{z}=-\frac{P_{2}{R_{2}}^{2}}{{R_{2}}^{2}-{R_{1}}^{2}}$$

The maximum stress concentration occurs at point M on the surface of the inner hole as shown in Figure 4c, $r=R_{1}$ in Equations (9) and (10), and it gives:

(12) $$\sigma_{\theta (\max)}=-\frac{2P_{2}R^{2}}{R^{2}-1}$$

(13) $$\sigma_{r}=0$$

(14) $$\sigma_{z}=-\frac{P_{2}R^{2}}{R^{2}-1}$$

where the wall-thickness ratio is $R=R_{2}/R_{1}$.

Circumferential stresses are generated at the hole periphery. For a closed cylindrical vessel with lateral holes subjected to internal pressure, Equation (12) can be interpreted as the unit stress $S_{x}$ in Equation (5):

(15) $$\sigma_{x}=-\frac{2P_{2}R^{2}}{R^{2}-1}\left(1+2\frac{b}{a}\right)$$

Since the cylinder is closed at both ends, axial stress develops as given by Equation (14). This axial stress component is interpreted as the unit stress $S_{y}$ in Equation (7):

(16) $$\sigma_{x}=\frac{P_{2}R^{2}}{R^{2}-1}$$

The stresses $\sigma_{x}$ given by Equations (15) and (16) both occur at point M in Figure 4c and are additive. Therefore, the total effective circumferential stress (i.e., the stress concentration circumferential stress) at point M (the minor axis location) of the cylinder is expressed as

(17) $$\sigma_{\theta \left|total\right.}=-\frac{2P_{2}R^{2}}{R^{2}-1}\left(1+2\frac{b}{a}\right)+\frac{P_{2}R^{2}}{R^{2}-1}$$

where the normal circumferential stress at M is as in Equation (12), and therefore, the stress concentration factor *K* at this location is

(18) $$K=\frac{-\frac{P_{2}R^{2}}{R^{2}-1}\left(1+4\frac{b}{a}\right)}{-\frac{2P_{2}R^{2}}{R^{2}-1}}=\frac{1+4\frac{b}{a}}{2}$$

Equation (18) can be used to calculate specific stress concentration factors based on the geometrical shape of the side hole. For a small circular side hole (*a = b*), the derived stress concentration factor reduces to *K* = 2.5 (Equation (18)).

Similarly, the total effective axial stress at the ends of the elliptical major axis can likewise be derived from Equations (6) and (8):

(19) $$\sigma_{z}=\frac{p_{0}R^{2}}{R^{2}-1}\left(1+2\frac{b}{a}\right)-\frac{2p_{0}R^{2}}{R^{2}-1}$$

The above analysis provides the fundamental basis for calculating other numerical values. To consider the size of the lateral hole, the expression for axial stress $\sigma_{z}$ can be further derived based on the net cross-sectional area of the load-bearing cylinder.

As shown in Figure 2, assume that the pressure length of the side hole is three-quarters of the hole length, which gives

(20) $$L=3/4\left(r_{0}-r_{i}\right)$$

According to the formula that stress equals load divided by area, it yields

(21) $$\sigma_{z}=\frac{\mathrm{force}}{\mathrm{net}\;\mathrm{area}}=\frac{{-P_{2}\pi r_{0}}^{2}}{\pi \left({R_{2}}^{2}-{R_{1}}^{2}\right)-2nr_{s}L}$$

For the case of a single hole, n = 1, it is known that

(22) $$\sigma_{z}=\frac{-P_{2}\pi R^{2}R_{s}}{\left(R-1\right)\left[\pi R_{s}\left(R+1\right)-1.5\right]}$$

where $r_{s}$ is the radius of the round hole, $R_{s}$ is the side hole ratio; round hole $R_{s}$ = $R_{1}/r_{s}$, elliptical hole $R_{s}$ = $R_{1}/a$. The above theoretical analysis demonstrates that, under internal pressure, a thick-walled cylinder with holes experiences stress concentration near the openings, and the local stresses significantly increases.

### 2.2. Leakage-Induced Stress Concentration of Vacuum Pressure Vessels

**Figure 5:**
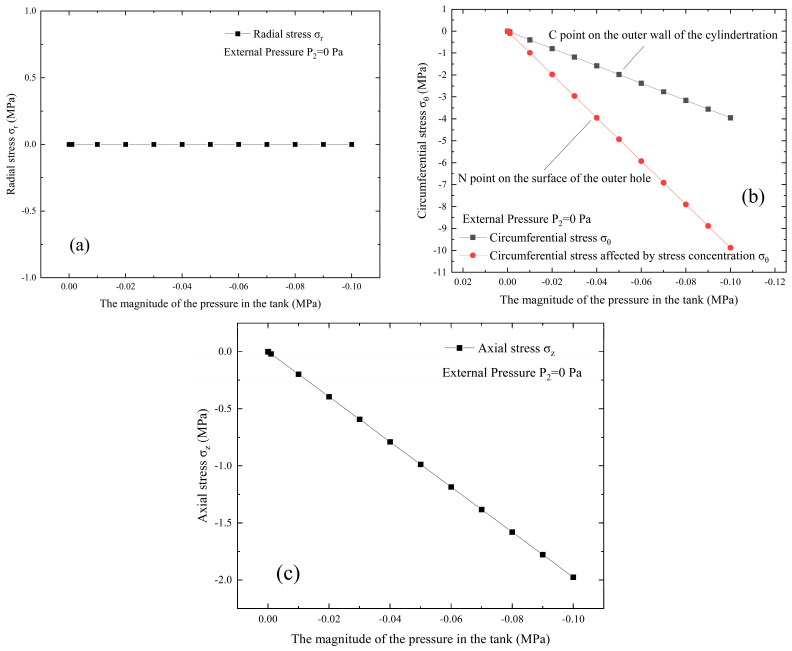
Stress variations in the vacuum tank with the gradual decrease in the internal pressure $P_{1}$ from 0 Pa to −0.1 MPa: (**a**) radial stress; (**b**) circumferential stress; (**c**) axial stress.

**Figure 6:**
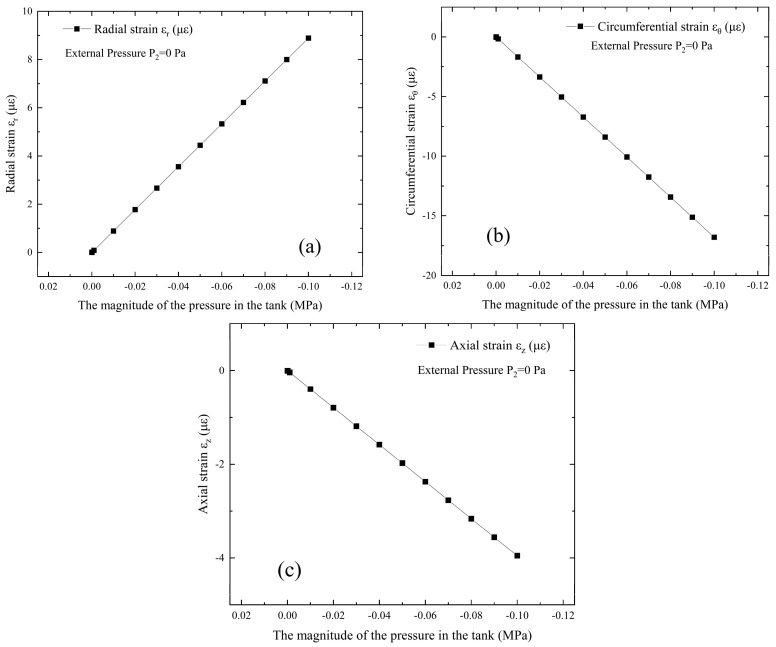
Strain variations in the vacuum tank with the gradual decrease in the internal pressure $P_{1}$ from 0 Pa to −0.1 MPa: (**a**) radial strain; (**b**) circumferential strain; (**c**) axial strain.

A vacuum tank with dimensions of ø400 mm × 700 mm and wall thickness of 5 mm is illustrated as an example, and the relevant parameters are summarized in Table 1. The stress–strain behavior of the vacuum tank is investigated under different conditions. The initial state of the vacuum tank corresponds to standard atmospheric pressure and a stress-free condition, which can be modeled as a thick-walled cylinder with neither internal pressure $P_{1}$ nor external pressure $P_{2}$. In the simulation of leakage without opening the valve, the vacuum extraction process corresponds to an external pressure $P_{2}$ maintained at zero, while the internal pressure $P_{1}$ decreases from 0 Pa to −0.1 MPa. The stress–strain distribution on the tank surface during this process is presented in Figure 5 and Figure 6. Point M in Figure 4c represents the inner hole surface, while the red line in Figure 5b indicates the stress change at point N in Figure 2 on the outer hole surface corresponding to point M. The black line indicates the stress change at point C in Figure 2, which is distant from the circular hole on the cylinder’s outer wall and unaffected by stress concentration.

Figure 5 illustrates the variations in radial, circumferential, and axial stresses of the vacuum tank, with internal pressure decreased from 0 Pa to −0.1 MPa. Figure 5b specifically presents the circumferential stress variations on the tank surface. The results indicate that, as the internal pressure drops from 0 Pa to −0.1 MPa, the radial stress remains at 0, the circumferential stress decreases from 0 MPa to −3.95 MPa, the stress-concentrated circumferential stress decreases from 0 MPa to −9.88 MPa, and the axial stress decreases from 0 MPa to −1.98 MPa, all consistent with mechanical theory.

By consulting relevant reference [40], the radial, circumferential, and axial strain components can be determined:

(23) $$\varepsilon_{r}=\frac{1}{E}\left(\left(1-2\mu \right)\frac{P_{1}{R_{1}}^{2}-P_{2}{R_{2}}^{2}}{{R_{2}}^{2}-{R_{1}}^{2}}-\left(1+\mu \right)\frac{\left(P_{1}-P_{2}\right){R_{1}}^{2}{R_{2}}^{2}}{{R_{2}}^{2}-{R_{1}}^{2}}\frac{1}{r^{2}}\right)$$

(24) $$\varepsilon_{\theta }=\frac{1}{E}\left(\left(1-2\mu \right)\frac{P_{1}{R_{1}}^{2}-P_{2}{R_{2}}^{2}}{{R_{2}}^{2}-{R_{1}}^{2}}+\left(1+\mu \right)\frac{\left(P_{1}-P_{2}\right){R_{1}}^{2}{R_{2}}^{2}}{{R_{2}}^{2}-{R_{1}}^{2}}\frac{1}{r^{2}}\right)$$

(25) $$\varepsilon_{z}=\frac{1}{E}\left(\left(1-2\mu \right)\frac{P_{1}{R_{1}}^{2}-P_{2}{R_{2}}^{2}}{{R_{2}}^{2}-{R_{1}}^{2}}\right)$$

Figure 6 can be obtained based on the above equation. Figure 6 presents the radial, circumferential, and axial strains of the vacuum tank, under gradually decreasing internal pressure from 0 Pa to −0.1 MPa. It can be observed that as the internal pressure decreases from 0 Pa to −0.1 MPa, the radial strain increases from 0 to 8.89 με, the circumferential strain decreases from 0 to −16.79 με, and the axial strain decreases from 0 to −3.95 με, all consistent with mechanical principles. Therefore, in subsequent experiments, physical states such as vacuum degree of the structure can be diagnosed based on the sensed strain information.

The stress–strain changes on the tank surface during vacuum extraction without opening the valve can simulate leakage. When leakage is simulated by opening the valve, it is assumed to be a small circular hole in the tank with a circumferential stress concentration factor of *K* = 2.5. Consequently, the circumferential stress in the concentration region is significantly higher than in other areas, resulting in correspondingly greater circumferential strain. According to related theories, stress concentration around the hole edge is not solely due to the slight reduction in the cross-sectional area. Even if the area is reduced by only a few percent compared to the non-porous state, stresses at the hole edge can increase several times. For holes with the same shape, the multiplicity of stress concentration is largely independent of the size of the hole, and then the influence of the hole size is ignored [42]. Stress concentration occurs around the hole and does not affect locations far from it. Stress quickly returns to nominal levels with increasing distance from the hole. Accordingly, this study focuses on stress concentration effects near the valve, while stress and strain variations farther away are referenced from Figure 5 and Figure 6.

## 3. Leakage Fault Identification Based on FBG Sensing Information

### 3.1. Experimental Investigation

For the stainless-steel vacuum tank, the vacuum extraction process can be modeled as that of a thick-walled cylinder subjected to constant external atmospheric pressure and a continuously decreasing internal pressure. During vacuum extraction, the internal pressure gradually decreases and can theoretically approach −0.1 MPa. This pressure differential induces compressive deformation of the tank wall. If no leakage occurs, the tank remains in a compressed state. If leakage occurs, the internal pressure rises until it equals the external pressure, thereby gradually relieving the compressive deformation and returning the tank to its initial stress-free state.

**Figure 7:**
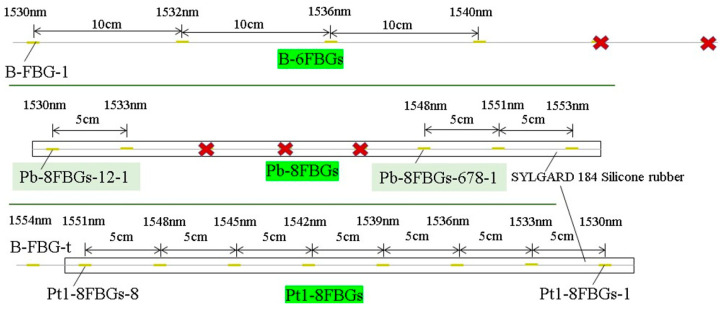
Arrangement of sensor points for vacuum process monitoring system for vacuum tank.

**Figure 8:**
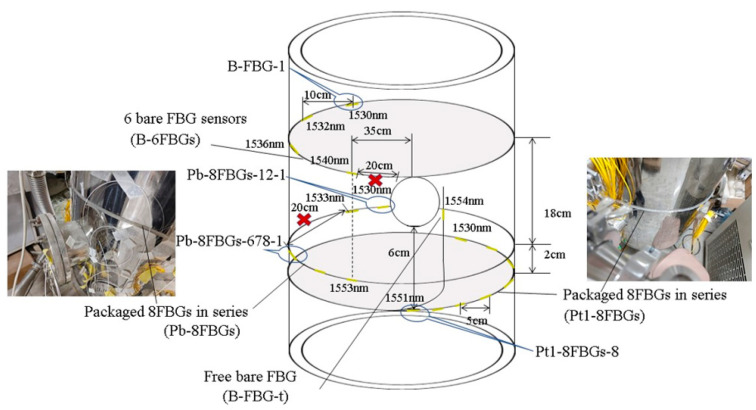
Sensor arrangement for vacuum process monitoring system of vacuum tank.

**Figure 9:**
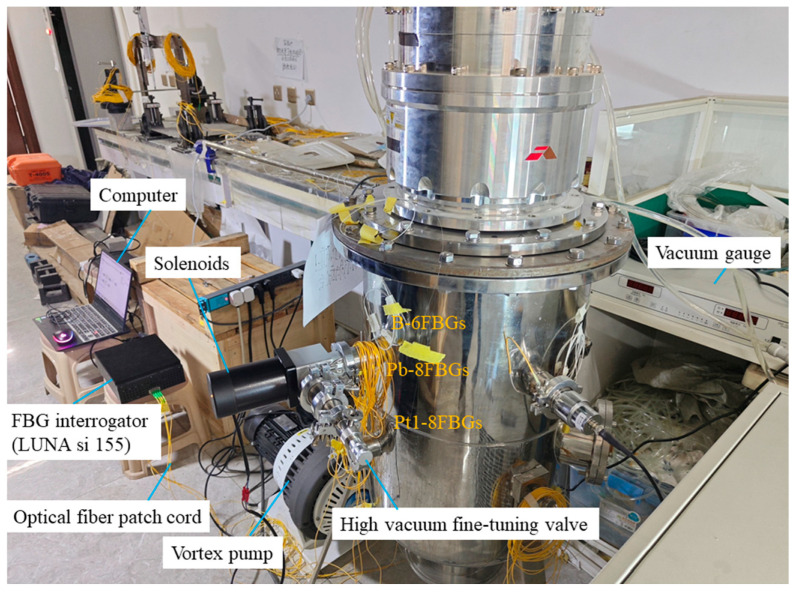
Physical photo of the monitoring system.

**Figure 10:**
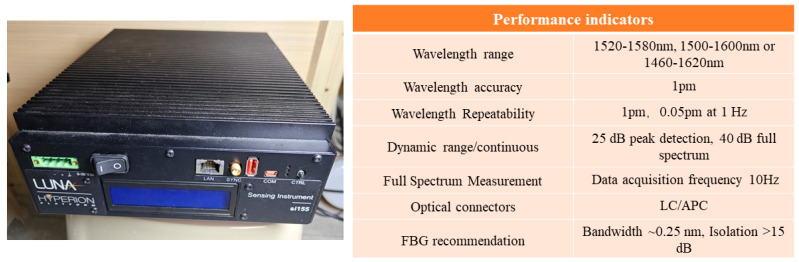
Physical photo and performance parameters of the optical fiber interrogator.

According to the valve positions and structural features of the vacuum tank, quasi-distributed FBG sensors were arranged near the valves in left/right clusters, with an additional upper and lower layer on the left side. During vacuum extraction, the vessel contracts under external pressure, while leakage induces pressure relief. To capture these strain variations, both bare FBG arrays in series (B-6FBGs) and flexible packaged FBG arrays in series (Pt1-8FBGs and Pb-8FBGs) were deployed. A bare FBG (B-FBG-t) was connected in series at the end of the Pt1-8FBGs to monitor vibration effects, positioned 6 cm above Pt1-8FBGs-6. The flexible packaged material is prepared by mixing SYLGARD 184 Silicone Elastomer Base and SYLGARD 184 Silicone Elastomer Curing Agent in a 10:1 ratio. To minimize deformation transmission losses caused by interlayer materials, the packaged FBG array was bonded to the vacuum tank wall by using the SYLGARD 184 Silicone Elastomer mixture. The sensor features a silicone rubber tube filled with SYLGARD 184 silicone mixture, with 3-mm diameter. The sensor design, layout, and physical monitoring system are shown in Figure 7, Figure 8 and Figure 9, while Figure 10 presents the parameters of the optical fiber interrogator si155. The si155 is built on the next-generation HYPERION platform, featuring groundbreaking capabilities, a high-performance DSP, and a real-time processing FPGA. The si155 enables rapid full-spectrum data acquisition. It incorporates an editable peak detection algorithm suitable for FBG, long-period grating (LPG), Fabry–Perot (FP), and Mach–Zehnder (M-Z) sensors, efficiently collecting data in closed-loop feedback applications.

### 3.2. Preliminary Data Analysis

To enhance monitoring accuracy, remove the vibration effect, a corresponding algorithm is proposed based on statistical data analysis. Three leakage tests under three operating conditions have been performed.

#### 3.2.1. Temperature Influence Test

**Figure 11:**
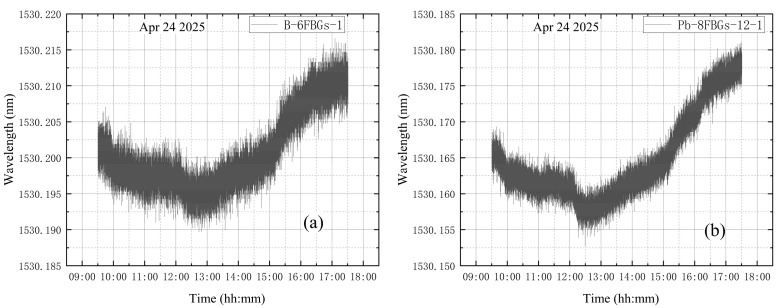
Wavelength variations: (**a**) B-6FBGs-1; (**b**) Pb-8FBGs-12-1.

On 24 April, measurements were taken from 09:30 to 17:30. During this period, the vacuum tank remained in its initial state under standard atmospheric pressure both inside and outside. The purpose of this test was to evaluate the influence of laboratory temperature. Figure 11 shows the wavelength variation in the first measurement point for the B-6FBGs and Pb-8FBGs. The variation was minimal, with a maximum amplitude of less than 0.01 nm over approximately 8 h. Therefore, the temperature effect can be considered negligible [43].

#### 3.2.2. Leak Monitoring Tests

**(1) No leakage in the whole experiment**

**Figure 12:**
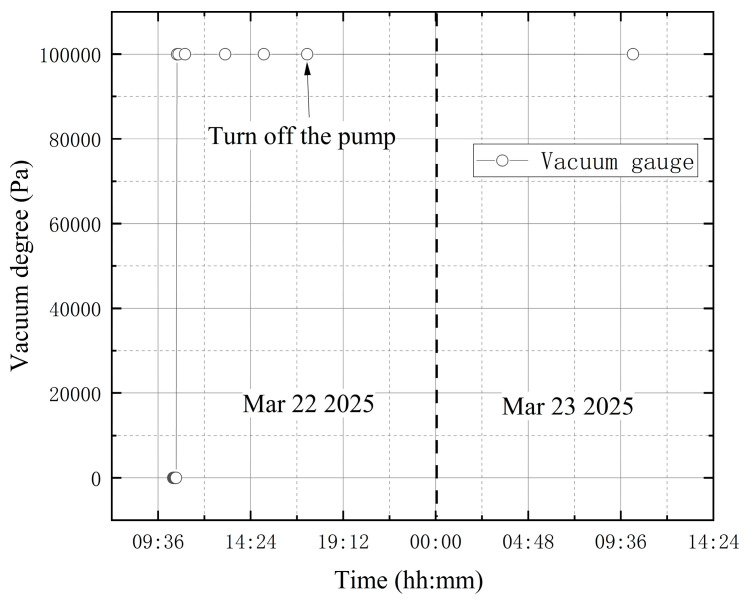
The change in vacuum degree in the first condition test.

Before activating the vortex pump, the high-vacuum fine-tuning valve was confirmed to be closed. On March 22, initial measurements were taken from 10:22 to 10:32, with the compound vacuum gauge reading 1 × 10^5^ Pa. The vortex pump was switched on at 10:33 for rough pumping, during which the gas in the bellows was extracted. The compound vacuum gauge indicated a decreasing tank pressure, reaching 26 Pa by 10:35. The valve was closed at 17:20, and the vortex pump was shut off, yielding a gauge reading of 2.3 × 10^−1^ Pa. On March 23 at 10:14, the gauge indicated 12 Pa. Without leakage, the tank pressure changed from 0.23 Pa to 12 Pa during prolonged storage, indicating that the experimental apparatus as a whole has a minute gap. Preliminary analysis suggests the gap may be located at the junction between the vacuum tank top and the molecular pump. However, since this location is distant from the tank wall monitored in the experiment and the leakage is extremely slow, the impact is negligible. The vacuum degree obtained from the vacuum gauge is shown in Figure 12. Since the vacuum degree $P_{v}$ is the atmospheric pressure minus the absolute pressure in the vacuum space, it thus gives

(26) $$P_{v}=P_{2}-\frac{E\frac{\Delta \lambda_{B}}{\lambda_{B}k_{\varepsilon }}\left({R_{2}}^{2}-{R_{1}}^{2}\right)+\left(1-2\mu \right)P_{2}{R_{2}}^{2}+\frac{\left(1+\mu \right)P_{2}{R_{1}}^{2}{R_{2}}^{2}}{r^{2}}}{\left(1-2\mu \right){R_{1}}^{2}+\frac{\left(1+\mu \right){R_{1}}^{2}{R_{2}}^{2}}{r^{2}}}$$

where $\lambda_{B}$ is the center wavelength of the FBG sensor, $\Delta \lambda_{B}$ is the center wavelength increment, and $k_{\varepsilon }$ is the strain sensitivity coefficient. It is worth noting that the value of $\lambda_{B}k_{\varepsilon }$ is approximately 1.2 pm/με [43].

**Figure 13:**
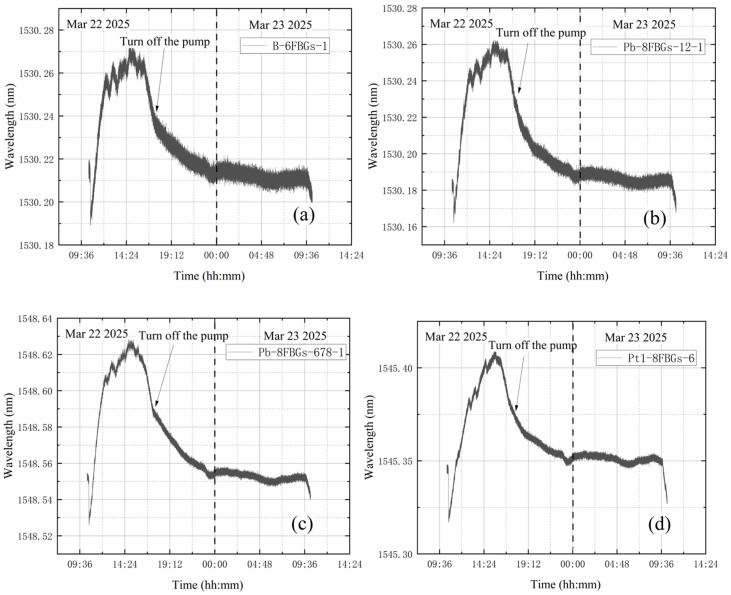
Wavelength variations in test without leakage: (**a**) B-6FBGs-1; (**b**) Pb-8FBGs-12-1; (**c**) Pb-8FBGs-678-1; (**d**) Pt1-8FBGs-6.

Figure 13 shows the wavelength variations over time from each sensor group. Pb-8FBGs-12 in Figure 13b refers to the first two points, and Pb-8FBGs-678 in Figure 13c refers to the last three points. The wavelength variation trends for the four designated sensor points are generally similar. A steep drop is observed at the beginning of the data. This is because after turning on the vortex pump for vacuum extraction, the internal pressure drops rapidly, with value below 10 Pa in 4–5 min, resulting in the shrinkage deformation and a wavelength decrease. The significant continuous decrease in the wavelength value is quite synchronized with the closing of the vortex pump. This process is not consistent with the theoretical derivation, and it is hypothesized that some external factor may have interfered with the monitoring results.

**(2) The high vacuum fine-tuning valve opened before vacuum extraction**

**Figure 14:**
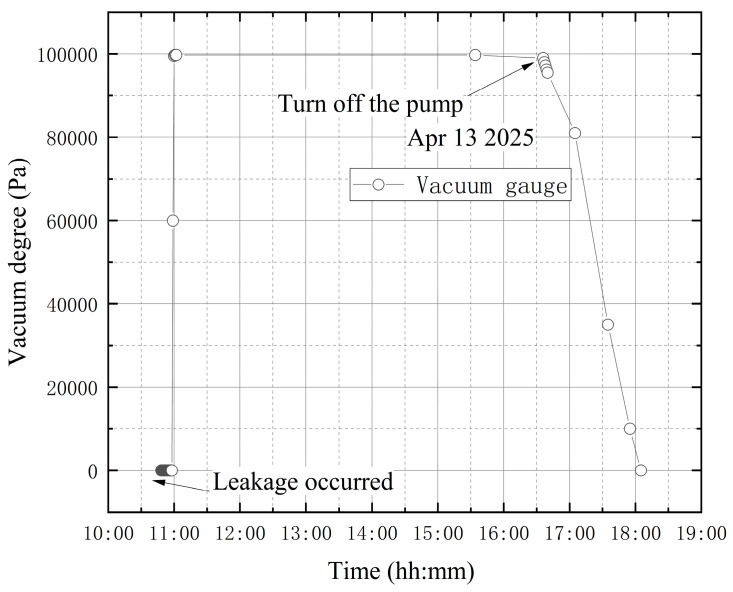
The change in vacuum degree in the first test of leakage condition.

The high-vacuum fine-tuning valve was opened counterclockwise by one full turn before starting the vortex pump. On 13 April, initial measurements were taken from 10:48 to 10:58, with the compound vacuum gauge reading 1 × 10^5^ Pa. The vortex pump was switched on at 10:58 for rough pumping, extracting gas from the bellows. Once the pre-stage valve was opened, the vortex pump began drawing gas from the vacuum tank through the connection between the bellows and the tank. The pre-stage valve connects the bellows to the vacuum tank. The compound vacuum gauge indicated a decreasing internal pressure, showing that the pumping speed exceeded the leakage flow rate. The readings were 4.0 × 10^4^ Pa at 10:59 and 2.6 × 10^2^ Pa at 11:02. The pumping speed then decreased until it equaled the leakage flow, stabilizing the internal pressure at approximately 2.6 × 10^2^ Pa. At 15:34, the gauge read 2.7 × 10^2^ Pa, and at 16:35 the pre-stage valve and vortex pump were closed. By 18:05, the gauge reading had returned to 1 × 10^5^ Pa, indicating that the tank had equilibrated with atmospheric pressure. The vacuum degree obtained according to the composite vacuum gauge reading is shown in Figure 14.

**Figure 15:**
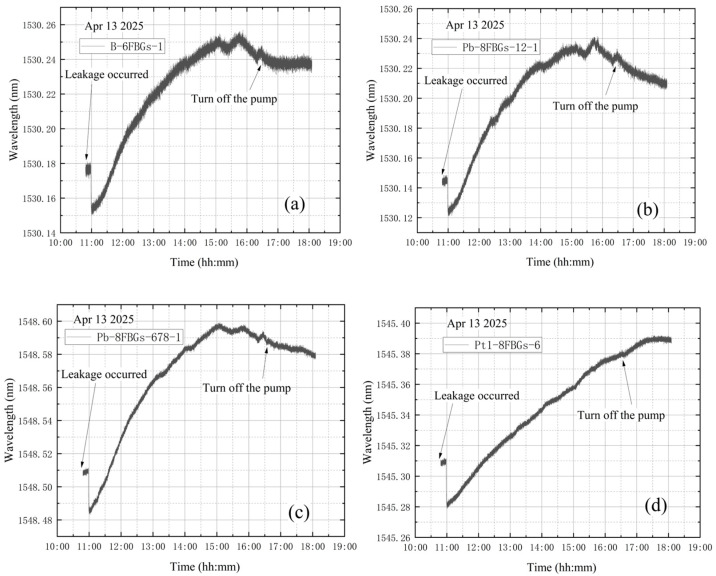
Wavelength variation in the first test of leakage condition: (**a**) B-6FBGs-1; (**b**) Pb-8FBGs-12-1; (**c**) Pb-8FBGs-678-1; (**d**) Pt1-8FBGs-6.

Figure 15 shows the wavelength variations from each sensor group. The wavelength variation trends for the four designated sensor points are generally similar. The shrinkage deformation of the tank makes the FBG sensors under compression, which leads to the wavelength reduction. However, the subsequent wavelength curves show a continuous increasing trend. The significant continuous decrease in the wavelength value is quite synchronized with the closing of the vortex pump. This experiment is to simulate leakage failure of a vacuum tank before vacuum extraction. The high vacuum trim valve is opened before the vortex pump is turned on to start the vacuum extraction process. The wavelength variation curves of the four measurement points in this experiment alone do not clearly show that leakage occurred before and during the whole process of vacuum extraction.

**(3) The high vacuum fine-tuning valve is opened at the end of vacuum extraction**

**Figure 16:**
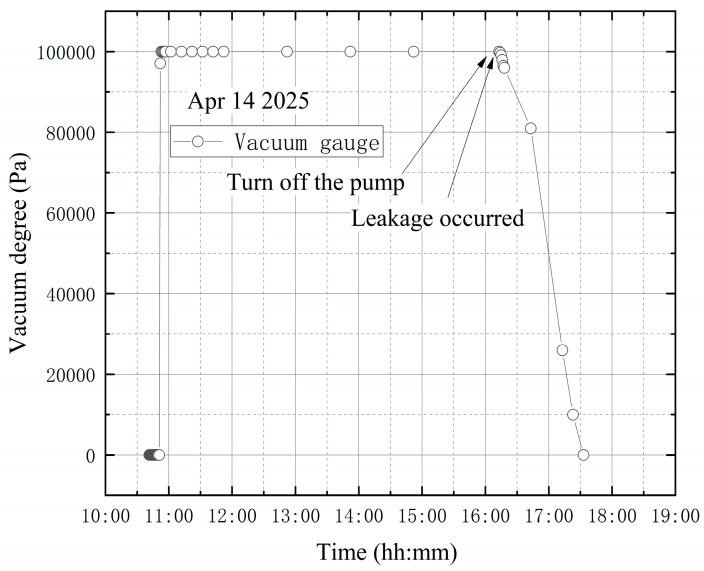
The change in vacuum degree in the second test of leakage condition.

The high-vacuum fine-tuning valve was kept closed before starting the vortex pump. On 14 April, initial measurements were taken from 10:41 to 10:51, with the compound vacuum gauge reading 1 × 10^5^ Pa. The vortex pump was switched on at 10:51 for rough pumping, extracting gas from the bellows. Once the pre-stage valve was opened, the pump began evacuating the vacuum tank. The compound vacuum gauge readings were 2.9 × 10^3^ Pa at 10:52 at 16:13. At this point, the pre-stage valve and vortex pump were closed, and the high-vacuum fine-tuning valve was opened by one full turn. The gauge then indicated 2.5 × 10^2^ Pa at 16:14 and 1 × 10^5^ Pa at 17:33, showing that the tank pressure had returned to atmospheric level. The vacuum degree image obtained according to the vacuum gauge reading is shown in Figure 16.

**Figure 17:**
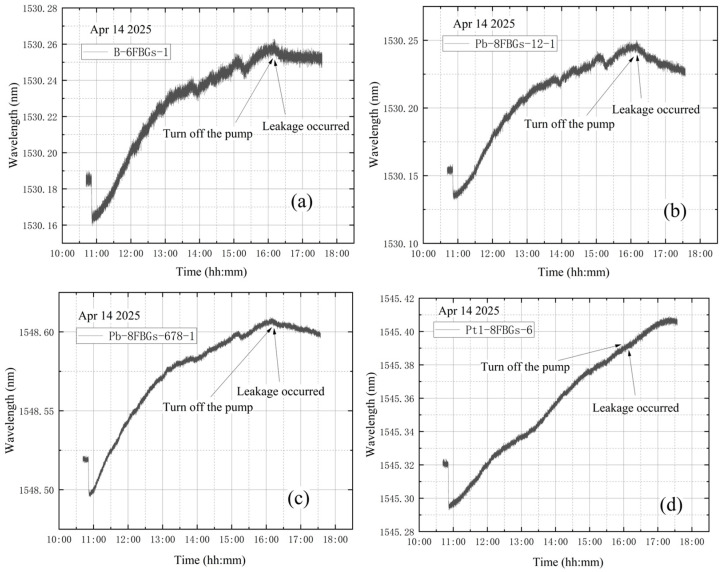
Wavelength variations in the second test of leakage condition: (**a**) B-6FBGs-1; (**b**) Pb-8FBGs-12-1; (**c**) Pb-8FBGs-678-1; (**d**) Pt1-8FBGs-6.

As shown in Figure 17, the wavelength variation trends at the four designated sensor points are generally similar. A steep drop in wavelength is observed at the beginning of the data. This occurs because, after turning on the vortex pump for vacuum extraction of the tank, the high-power operation of the pump can reduce the internal pressure below 10 Pa within 4–5 min. The rapid pressure drop causes shrinkage deformation of the vacuum tank, leading to a decrease in wavelength. Subsequently, the wavelength curves exhibit a continuous increasing trend. The significant continuous decrease in the wavelength value is quite synchronized with the closing of the vortex pump. This experiment simulated a leakage failure of the vacuum tank in a near-vacuum state, with the high-vacuum fine-tuning valve opened after vacuum extraction. The wavelength variation curves at the four measurement points do not clearly indicate that leakage occurred either before or during the entire vacuum extraction process.

**(4) The high vacuum fine-tuning valve is opened during vacuum extraction**

**Figure 18:**
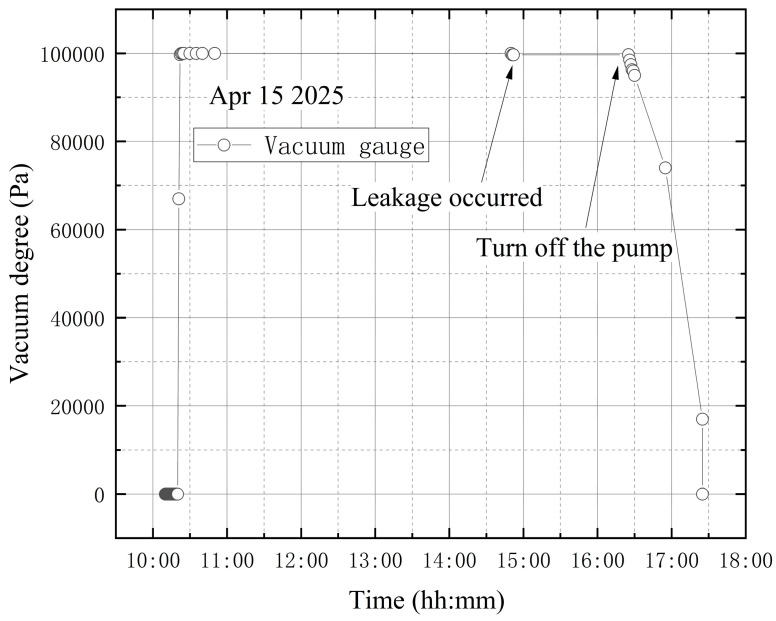
The change in vacuum degree in the third leakage condition test.

The high-vacuum fine-tuning valve was confirmed to be closed before starting the vortex pump. On 15 April, initial measurements were taken from 10:10 to 10:20. The vortex pump was then activated at 10:20 for rough pumping, extracting gas from the bellows. Once the pre-stage valve was opened, the vortex pump began evacuating the vacuum tank, causing the internal pressure to decrease. The compound vacuum gauge readings were 3.3 × 10^4^ Pa at 10:21 and 5.7 × 10^−1^ Pa at 14:50. At this stage, the high-vacuum fine-tuning valve was opened counterclockwise by one full turn. The gauge indicated 3.2 × 10^2^ Pa at 14:51 and 3.3 × 10^2^ Pa at 14:52. The pumping speed then increased until it balanced with the leakage flow rate through the fine-tuning valve, stabilizing the internal pressure at approximately 3.3 × 10^2^ Pa. Finally, at 17:35, the gauge reading returned to 1 × 10^5^ Pa, showing that the tank had reached atmospheric pressure. The vacuum degree image obtained according to the vacuum gauge reading is shown in Figure 18.

**Figure 19:**
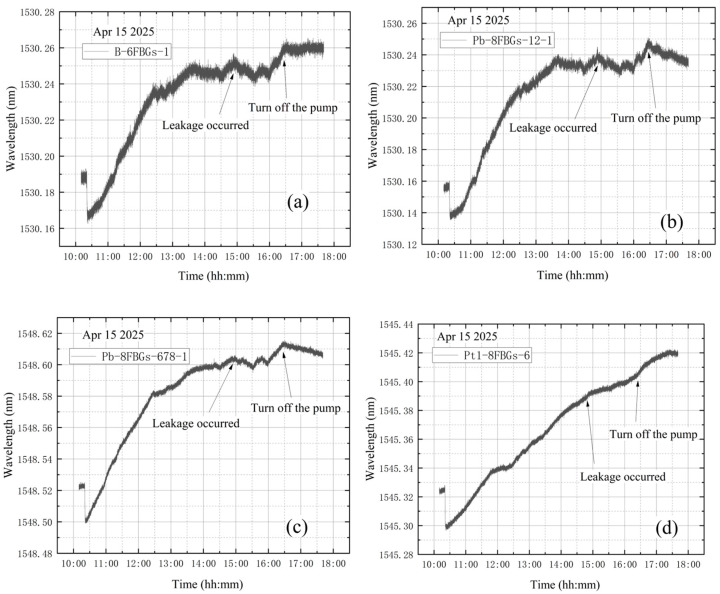
Wavelength variations in the third leakage condition test: (**a**) B-6FBGs-1; (**b**) Pb-8FBGs-12-1; (**c**) Pb-8FBGs-678-1; (**d**) Pt1-8FBGs-6.

As shown in Figure 19, the wavelength variation trends at the four designated sensor points are generally similar. A steep drop in wavelength is observed at the beginning of the data. This occurs because, after turning on the vortex pump for vacuum extraction of the tank, the internal pressure drops rapidly, causing shrinkage deformation of the vacuum tank and a decrease in wavelength. Subsequently, the wavelength curves exhibit a continuous increasing trend. The significant continuous decrease in the wavelength value is quite synchronized with the closing of the vortex pump. To simulate a leakage failure of the vacuum tank during vacuum extraction, the high-vacuum fine-tuning valve was opened during the process. The wavelength variation curves do not clearly indicate that leakage occurred either before or throughout the vacuum extraction.

Comparative analysis of the data from the four tests—the simulated non-leakage test and the three leakage tests—indicates that the wavelength data of the non-leakage test are essentially the same as those of the three leakage tests before the pump shutdown. The wavelength in the non-leakage test gradually decreases, which is consistent with the theoretical derivation that the wavelength should slightly decrease or remain unchanged during the vacuum extraction phase [44,45,46]. In reality, the wavelength curve exhibits a continuous increasing trend, which is attributed to the vibration effect during pump operation that interferes with the wavelength variation. Comparing the wavelength data of the three leakage tests, it can be seen that the trends at each measurement point are generally consistent, making it difficult to determine the occurrence of leakage. Considering that the vibration effect interferes with the wavelength variations, subsequent attempts were made to perform vibration correction to further observe and analyze the wavelength changes under each operating condition.

### 3.3. Vibration Influence Analysis

**Figure 20:**
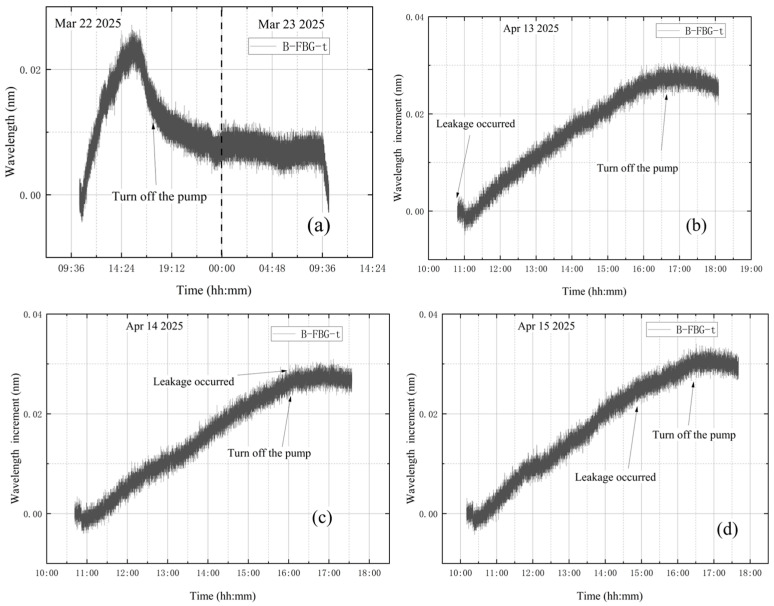
Wavelength incremental variations in B-FBG-t: (**a**) no leakage; (**b**) leakage before vacuum extraction; (**c**) leakage after vacuum extraction; (**d**) leakage during vacuum extraction.

The wavelength variation curves under the four test conditions show that, once the vortex pump is activated, vibration induces a continuous upward trend in the wavelength data. When the pump is shut down, this trend stops and the data begin to decrease, confirming that the vibration originates from the pump. Preliminary analysis of the experimental data indicates that vibration has a significant impact on measurement accuracy. To address this, the wavelength data recorded by the dedicated vibration-monitoring sensor B-FBG-t were used for correction. Four sets of vibration data were obtained from the four tests, as shown in Figure 20.

As shown in Figure 20, the graphs illustrate the wavelength increment variations in B-FBG-t under the four conditions. The variation trends closely match those observed at other measurement points affected by vibration in the initial experiments. Notably, no initial steep drop occurred during the process. This indicates that the wavelength increment of B-FBG-t can reliably represent the vibration effect. Therefore, further optimization and correction are required to improve measurement accuracy and ensure that the results more faithfully reflect the actual vibration.

Through systematic statistical analysis of a large dataset, and by accounting for factors that could affect the results, the average correction factor was determined. This procedure ensures data reliability and analytical accuracy, enabling a substantial reduction or even elimination of vibration effects. It also provides robust data support and a theoretical basis for subsequent research and decision-making using the corrected results.

To maximize correction accuracy, the sixth measurement point of the sensor Pt1-8FBGs—located closest to the vibration-monitoring sensor B-FBG-t—was selected as the reference for vibration correction. The slope $k_{1}$ was calculated by subtracting the minimum wavelength increment value after the steepest drop from the first maximum value at Pt1-8FBGs-6, then dividing by time. Since B-FBG-t measured only vibration, no steep drop was observed at the beginning of vacuum extraction. Therefore, the slope $k_{2}$ was obtained by dividing the first maximum wavelength increment by time. The minimum criterion for selecting $k_{1}$ is the lowest wavelength recorded during the initial vacuum extraction. The criterion for selecting the minimum value of $k_{2}$ is that since the vibration measurement points do not monitor tank strain, it is initially set to zero. Subsequently, due to vibration effects, the wavelength gradually increases. The maximum criterion for selecting $k_{1}$ and $k_{2}$ is the highest wavelength measured before this increasing trend ceases. From this, the correction factor $k_{0}$ can be obtained:

(27) $$k_{0}=\frac{k_{1}}{k_{2}}$$

**Figure 21:**
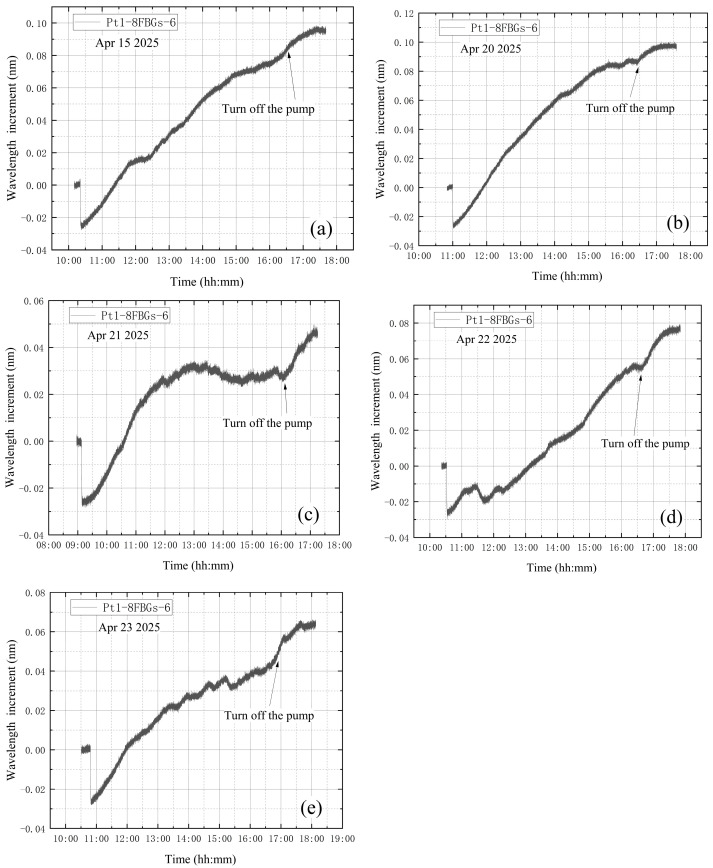
The wavelength increment of Pt1-8FBGs-6 before correction: (**a**) the first test; (**b**) the second test; (**c**) the third test; (**d**) the fourth test; (**e**) the fifth test.

**Figure 22:**
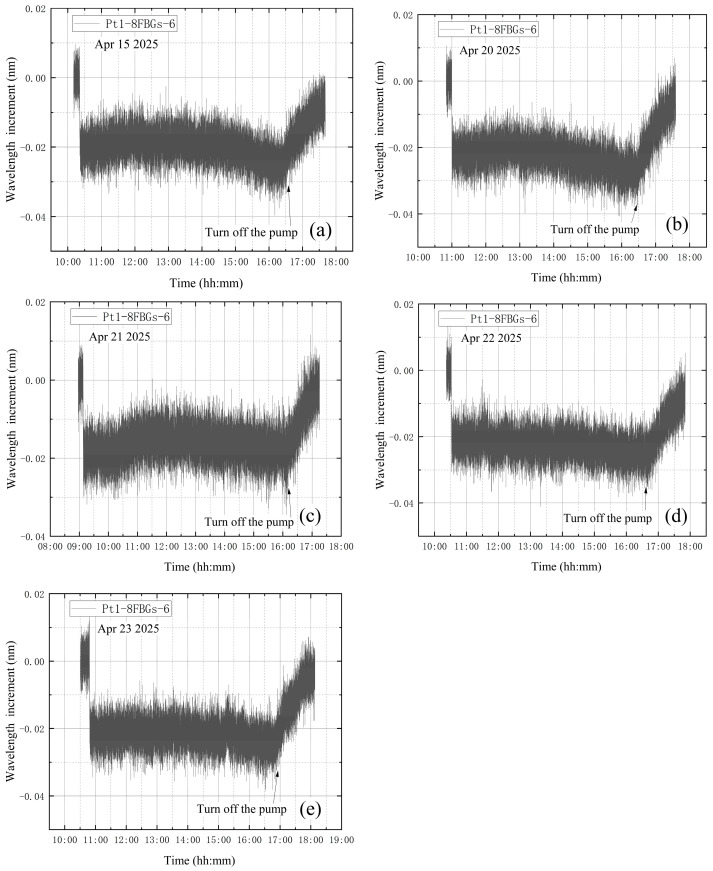
Modified wavelength increments of Pt1-8FBGs-6: (**a**) the first test; (**b**) the second test; (**c**) the third test; (**d**) the fourth test; (**e**) the fifth test.

The average value of the correction factor $k_{0}$ of the five experiments is obtained as $\bar{k_{0}}$. $\bar{k_{0}}$ for the leakage condition during vacuum extraction is 3.51. After calculation, the standard error is obtained as 0.111, with a 95% confidence interval of [3.21, 3.82]. Multiply the wavelength increment of B-FBG-t by the average value of the correction factor $\bar{k_{0}}$ to obtain the corrected vibration data, and subtract the corrected vibration data from the wavelength increment of Pt1-8FBGs-6 to obtain the vibration-corrected wavelength increment of Pt1-8FBGs-6. The wavelength increments of Pt1-8FBGs-6 before vibration correction in five experiments are shown in Figure 21. The vibration-corrected Pt1-8FBGs-6 wavelength increments for the five experiments are shown in Figure 22.

Figure 22 shows the incremental wavelength variation in Pt1-8FBGs-6 under the leakage condition during vacuum extraction. The incremental wavelength curves change after the vacuum extraction and pump shutdown phases, except for a steep drop at the beginning of vacuum extraction. Theoretically, after a leak occurs during vacuum extraction, the internal pressure rises from about 0.5 Pa to approximately 300 Pa within 3 min and then remains essentially stable. However, this pressure change has only a minor effect on the stress and strain of the tank wall, so the wavelength increment should remain essentially constant during the intermediate vacuum extraction phase. After the vortex pump is turned off, the vacuum tank continues to leak, leading to a gradual increase in internal pressure until it returns to standard atmospheric pressure.

Vibration correction has significantly improved the accuracy of the measurement results. Specifically, the deviation between the wavelength increment after vibration correction and the theoretically derived value at each measurement point has been markedly reduced, and the consistency between the two has been greatly enhanced. This result strongly demonstrates the effectiveness of the vibration correction method, indicating that it can efficiently eliminate vibration interference in the measurements. The introduction of vibration correction steps substantially improves the reliability and accuracy of the measurement system, providing a more precise data foundation for subsequent research. It not only improves the accuracy of the measurements but also enhances the repeatability and reliability of the experimental results.

## 4. Leakage Identification After Vibration Correction

**Figure 23:**
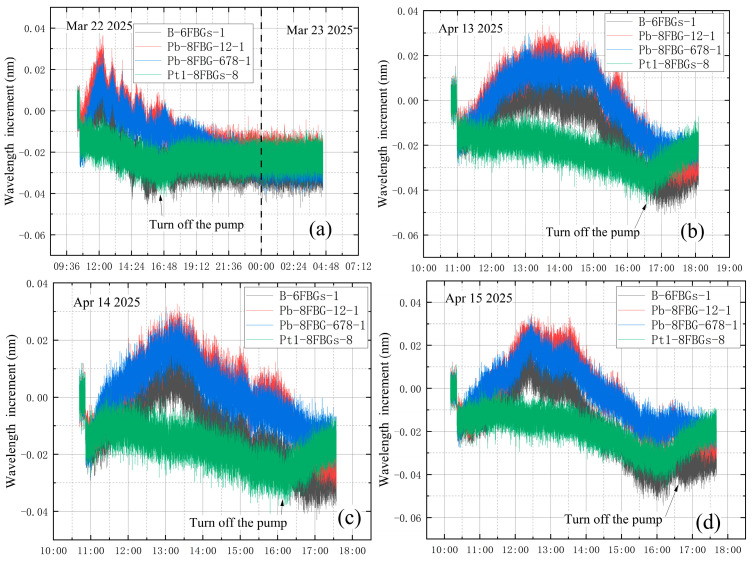
Vibration-corrected wavelength increments of sensors: (**a**) no leakage; (**b**) leakage before vacuum extraction; (**c**) leakage at the end of vacuum extraction; (**d**) leakage during vacuum extraction.

**Figure 24:**
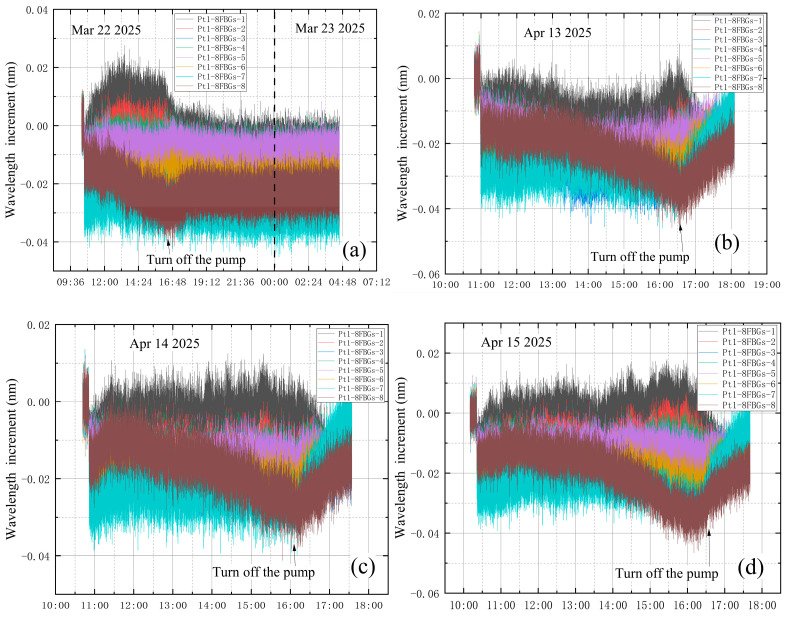
Vibration-corrected wavelength increments of Pt1-8FBGs: (**a**) no leakage; (**b**) leakage before vacuum extraction; (**c**) leakage at the end of vacuum extraction; (**d**) leakage during vacuum extraction.

The vibration-corrected test data were analyzed under four conditions: no leakage and three leakage scenarios. Figure 23 shows the wavelength increments of different sensors after vibration correction under these four conditions. As shown in Figure 23, since the correction coefficients are obtained based on the measured data from Pt1-8FBGs sensors, the corrected data of Pt1-8FBGs align more closely with theoretical predictions, indicating that the correction is effective. To better analyze the effect of vacuum leakage, the vibration-corrected wavelength increments of the eight measurement points on sensor Pt1-8FBGs under the four conditions are plotted in Figure 24.

Figure 24 shows that the eight measurement points on Pt1-8FBGs closest to the high-vacuum fine-tuning valve exhibit significant differences in incremental wavelength variations due to their distances from the valve. The eighth measurement point of Pt1-8FBGs is the closest, located 6 cm from the valve. The distances then increase sequentially, with the first measurement point of Pt1-8FBGs being the farthest.

To identify leakage through the FBG sensors and to determine whether the leakage location can be inferred from the stress concentration phenomenon, only the eight measurement points on the Pt1-8FBGs sensors closest to the high-vacuum fine-tuning valve are selected for analysis. For a more intuitive analysis, the wavelength increments are converted to vacuum levels for subsequent evaluation, based on previous studies [40].

### 4.1. No Leakage During the Whole Experiment

**Figure 25:**
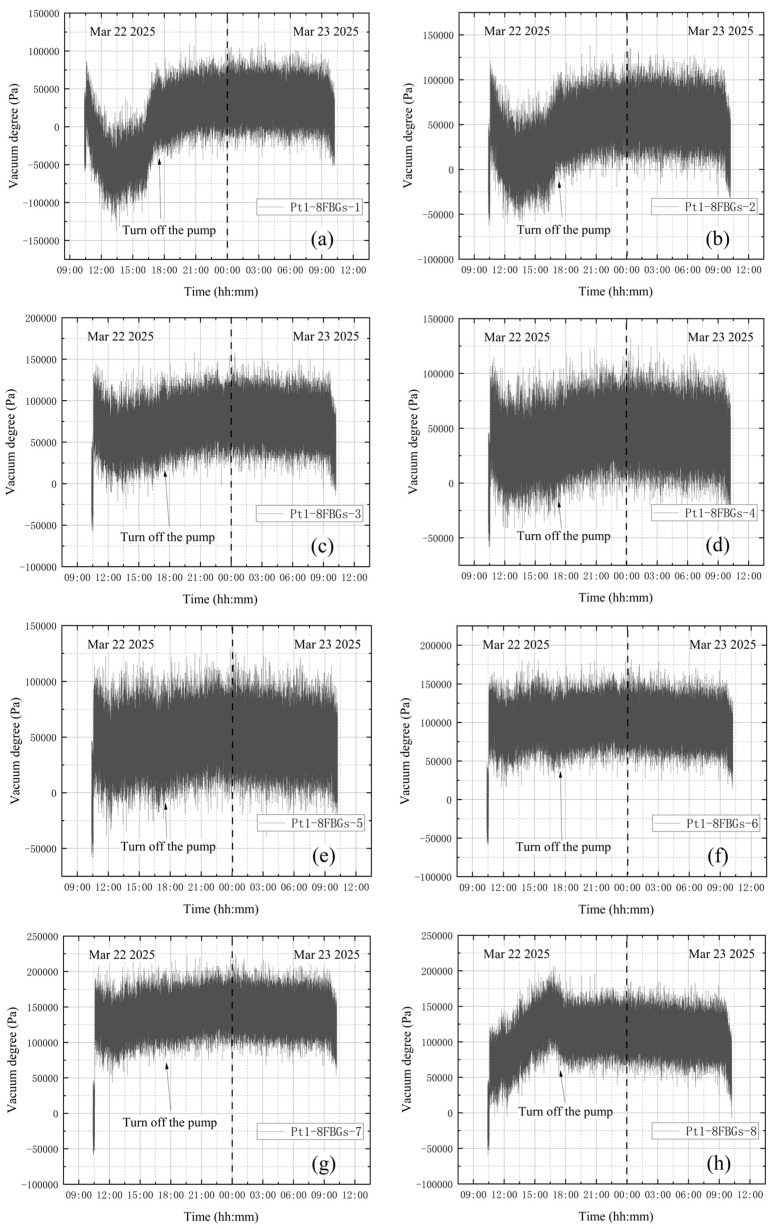
Vacuum degree measured by Pt1-8FBGs: (**a**) Pt1-8FBGs-1; (**b**) Pt1-8FBGs-2; (**c**) Pt1-8FBGs-3; (**d**) Pt1-8FBGs-4; (**e**) Pt1-8FBGs-5; (**f**) Pt1-8FBGs-6; (**g**) Pt1-8FBGs-7; (**h**) Pt1-8FBGs-8.

Vibration correction is applied to the data measured at the eight points of the sensor Pt1-8FBGs without leakage, and the modified data are converted to vacuum degree by using Equation (26). Figure 25 shows the change in vacuum degree measured by Pt1-8FBGs after vibration correction. As shown in Figure 25, due to the relatively large distance from B-FBG-t, the vibration correction effects of Pt1-8FBG-1 and Pt1-8FBG-2 are relatively poor. According to the vacuum degrees reflected from Pt1-8FBG-6, Pt1-8FBG-7 and Pt1-8FBG-8, the vacuum degree increases rapidly at the beginning of vacuum extraction, and then tends to be stabilized after it grows up to 1 × 10^5^ Pa. The stable state is maintained until the end of the experiment, which indicates that no leakage occurred during the whole experiment, which is also consistent with the actual situation, indicating that the vibration correction is effective.

### 4.2. Leakage Before Vacuum Extraction

**Figure 26:**
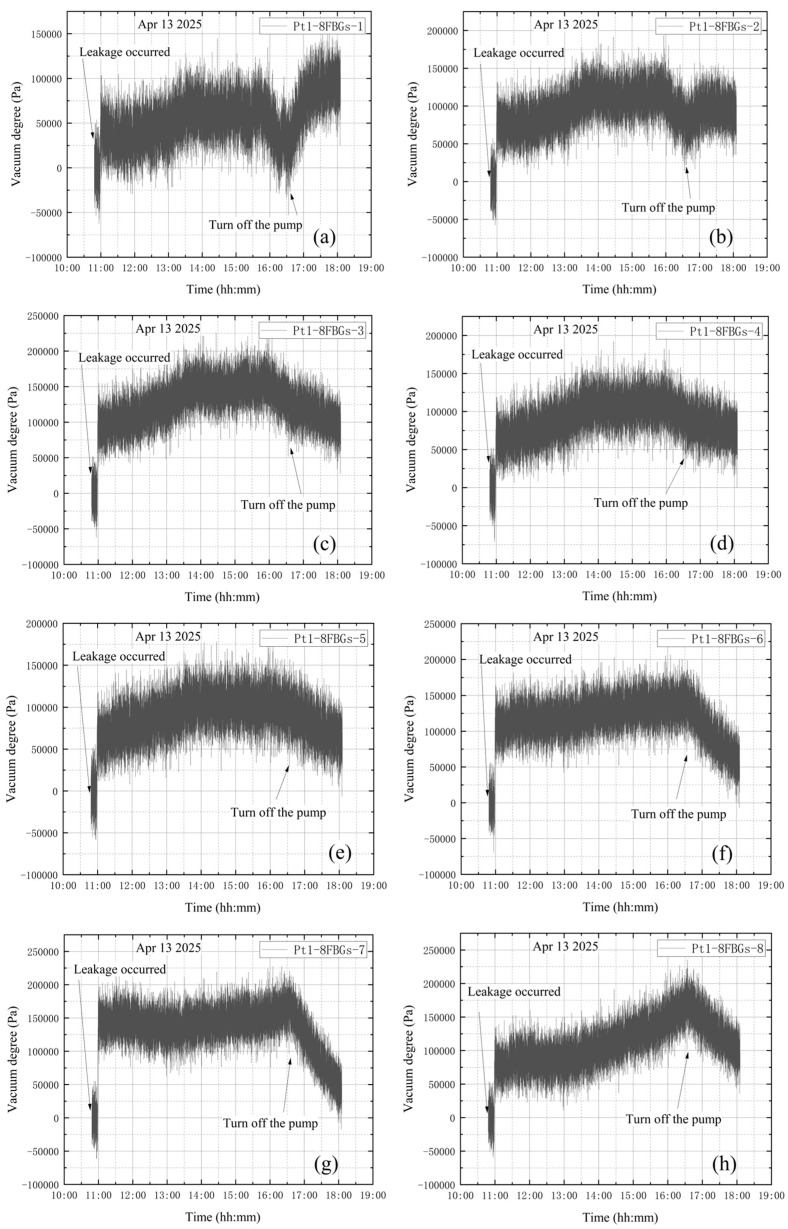
Vacuum degree measured by Pt1-8FBGs: (**a**) Pt1-8FBGs-1; (**b**) Pt1-8FBGs-2; (**c**) Pt1-8FBGs-3; (**d**) Pt1-8FBGs-4; (**e**) Pt1-8FBGs-5; (**f**) Pt1-8FBGs-6; (**g**) Pt1-8FBGs-7; (**h**) Pt1-8FBGs-8.

Figure 26 shows the change in vacuum level measured by the Pt1-8FBGs sensor after vibration correction under the leakage condition before vacuum extraction.

The valve was already open before the vortex pump was started, resulting in stress concentrations near the valve throughout the experiment. While the vortex pump is running, the internal pressure initially decreases and then stabilizes, which is generally consistent with theoretical predictions. However, after shutting down the vortex pump, comparison with Figure 25 shows that leakage occurs in the tank. After closing the pre-stage valve and the vortex pump at 16:35, a significant drop in vacuum level was observed, indicating the occurrence of leakage. Measurement points farther from the valve drop sharply due to the increase in internal pressure until reaching zero. In contrast, for the measurement point Pt1-8FBG-8, which is close to the valve, the vacuum level decreases significantly less than at other points. This is attributed to stress concentration near the valve, where stress changes more slowly than at other locations due to the increased internal pressure.

### 4.3. Leakage at the End of Vacuum Extraction

**Figure 27:**
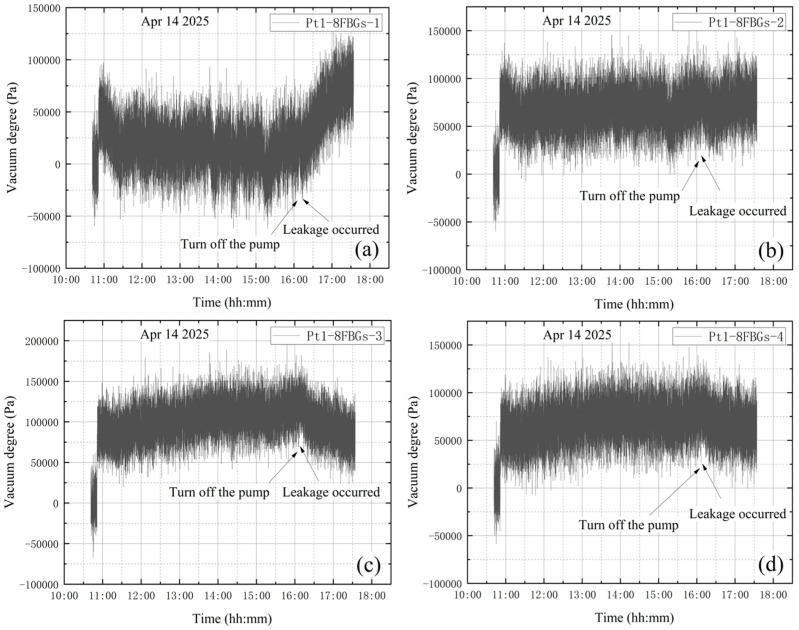

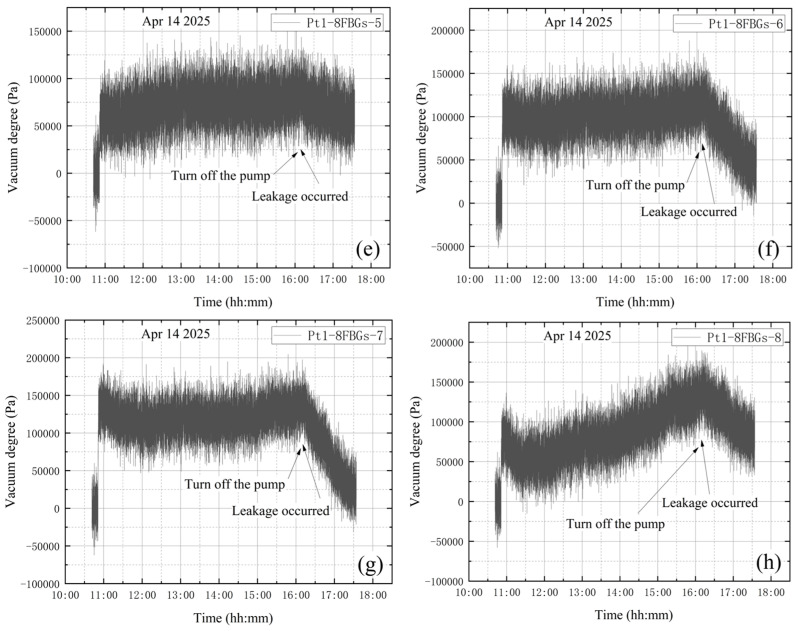
Vacuum degree measured by Pt1-8FBGs: (**a**) Pt1-8FBGs-1; (**b**) Pt1-8FBGs-2; (**c**) Pt1-8FBGs-3; (**d**) Pt1-8FBGs-4; (**e**) Pt1-8FBGs-5; (**f**) Pt1-8FBGs-6; (**g**) Pt1-8FBGs-7; (**h**) Pt1-8FBGs-8.

Figure 27 shows the change in vacuum level measured by the Pt1-8FBGs sensor after vibration correction under the leakage condition at the end of vacuum extraction. The changes in Figure 27 after the pump is turned off indicate that the tank is already leaking at this point. When the vortex pump is turned off at 16:13 and the high-vacuum fine-tuning valve is opened, the internal pressure gradually increases and then stabilizes, causing a gradual decrease in vacuum level. Measurement points farther from the valve detect a sharp drop in vacuum level due to the pressure increase until reaching zero. In contrast, compared with Pt1-8FBG-6 and Pt1-8FBG-7, the decrease in vacuum level at the measurement point adjacent to the valve, Pt1-8FBG-8, is significantly smaller due to the pronounced effect of stress concentration. This is attributable to stress concentration near the valve, where stress changes slowly in response to the increasing internal pressure. These observations indicate that leakage occurs in the vicinity of measurement point Pt1-8FBG-8.

### 4.4. Leakage During Vacuum Extraction

**Figure 28:**
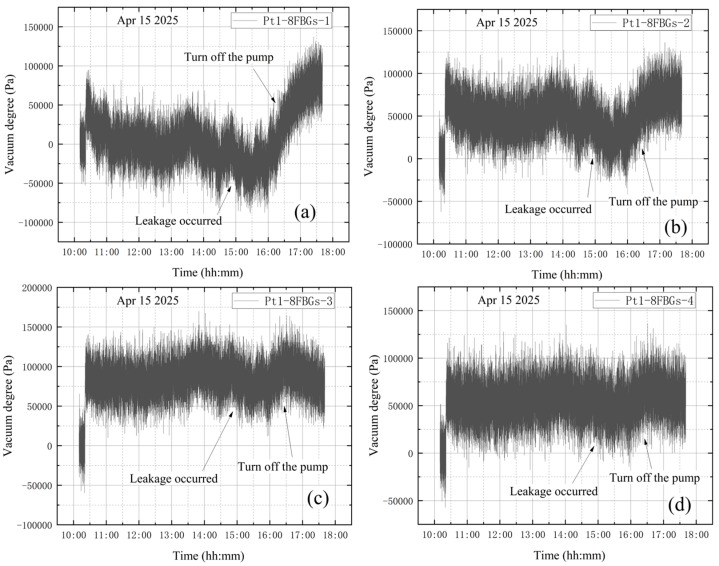

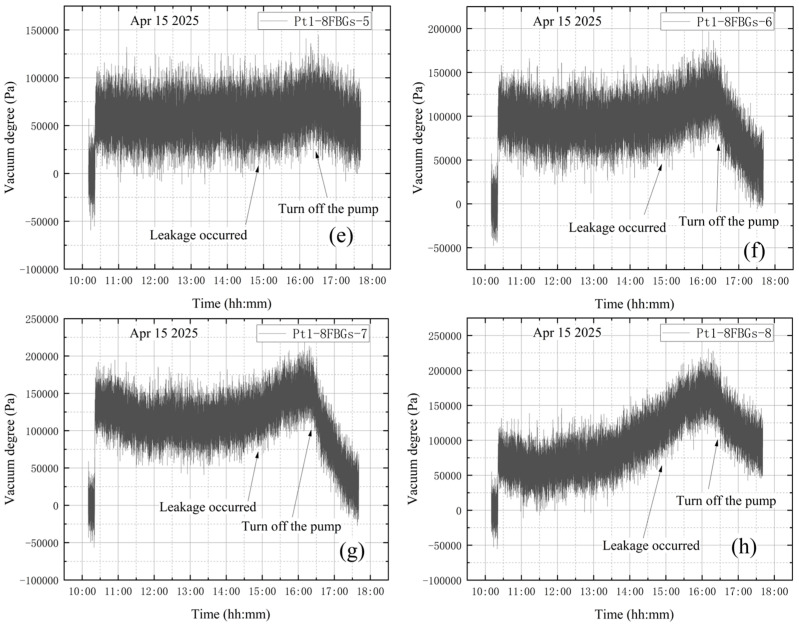
Vacuum degree measured by Pt1-8FBGs: (**a**) Pt1-8FBGs-1; (**b**) Pt1-8FBGs-2; (**c**) Pt1-8FBGs-3; (**d**) Pt1-8FBGs-4; (**e**) Pt1-8FBGs-5; (**f**) Pt1-8FBGs-6; (**g**) Pt1-8FBGs-7; (**h**) Pt1-8FBGs-8.

Figure 28 shows the change in vacuum level measured by the Pt1-8FBGs sensor after vibration correction under the leakage condition during vacuum extraction.

When the high-vacuum fine-tuning valve is opened at 14:50 during vacuum extraction, the pressure inside the tank gradually increases and then tends to stabilize. Figure 28c,d show that the vacuum level in the tank decreases, indicating that the vacuum level data monitored by the FBG sensor can reveal vacuum leakage. After shutting down the pump, the suspected leakage location can also be identified through analysis based on stress concentration theory. Following the shutdown of the pre-stage valve and vortex pump at 16:25, the vacuum level decreases sharply until reaching zero. Compared with the data from Pt1-8FBG-6 and Pt1-8FBG-7, the vacuum level at Pt1-8FBG-8, adjacent to the valve, decreases significantly less due to the pronounced stress concentration effect. This is attributed to stress concentration near the valve, where stress changes more slowly than at other locations due to the increased internal pressure. These observations are highly consistent with theoretical derivations, further confirming the applicability and accuracy of stress concentration theory in the experimental setup. Additionally, this highlights the importance of accurately monitoring and evaluating stress concentration phenomena under similar experimental conditions.

In summary, vibration correction has a significant effect on the three measurement points Pt1-8FBG-6, Pt1-8FBG-7, and Pt1-8FBG-8. Further analysis indicates that the relative position between the measurement point and the vibration reference point is the key factor affecting the correction effect. Specifically, the vibration reference point is closest to Pt1-8FBG-6, so the influence of vibration interference is most direct and pronounced. In contrast, the vibration correction effect at Pt1-8FBG-7 and Pt1-8FBG-8 gradually diminishes due to their greater distance from the vibration source. This demonstrates that, in practical engineering applications, the layout of measurement points and the geometry of the structure have a significant influence on the distribution and effectiveness of vibration correction, which should be fully considered during experimental design. These results strongly indicate that the combination of FBG sensing technology and stress concentration theory can achieve effective analysis and localization of leakage, providing a promising and practical method for leakage detection in engineering applications.

### 4.5. Construction of Fitting Equation

**Figure 29:**
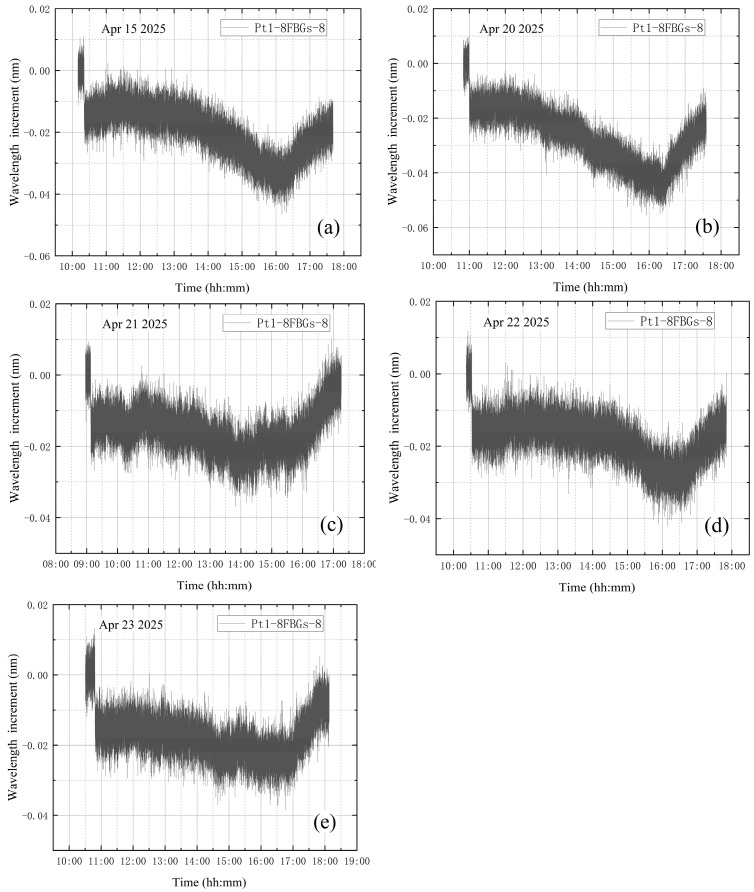
Modified wavelength increments of Pt1-8FBGs-8: (**a**) the first test; (**b**) the second test; (**c**) the third test; (**d**) the fourth test; (**e**) the fifth test.

To explain the variation in wavelength increments under leakage conditions during vacuum extraction, five experimental datasets, with the high-vacuum fine-tuning valve opened during vacuum extraction, are analyzed. The wavelength increments of Pt1-8FBGs-8 are shown in Figure 29. The final fitting equation is obtained using the piecewise function method combined with the weighted average method.

Firstly, the interval division of the piecewise function is carried out to ensure that each interval accurately reflects the changing characteristics of the data. Based on the operating conditions and experimental images of each experiment, the data are divided into three parts: (1) the steep drop in wavelength increment within a few minutes after the vortex pump is turned on. This segment is nearly vertical, and the steep drop is evident, so no fitting equation is applied to this section; (2) the downward trend following the initial sharp drop until the vortex pump is turned off. This segment can be fitted with a nonlinear equation; (3) the subsequent growth trend until the end after the vortex pump is turned off. This segment can be fitted using a linear equation.

The second curve from the five experiments is then fitted to obtain five nonlinear equations, which are

(28) $$\begin{array}{l} y_{1}=-8\cdot 10^{-11}{x_{1}}^{2}+6\cdot 10^{-7}x_{1}-0.0138\\ y_{2}=-6\cdot 10^{-11}{x_{2}}^{2}+4\cdot 10^{-7}x_{2}-0.0145\\ y_{3}=-5\cdot 10^{-11}{x_{3}}^{2}+4\cdot 10^{-7}x_{3}-0.0139\\ y_{4}=-7\cdot 10^{-11}{x_{4}}^{2}+9\cdot 10^{-7}x_{4}-0.0168\\ y_{5}=-8\cdot 10^{-12}{x_{5}}^{2}-3\cdot 10^{-7}x_{5}-0.0144 \end{array}$$

Note that in the above five equations, the time when *x_i_* (i = 1, 2…5) equals 0 refers to one minute after the vortex pump is turned on. Using the weighted average method, the five quadratic function equations are averaged. For each coefficient in the quadratic function equation (quadratic term, linear term, and constant term), the weighted average is calculated separately, and the final quadratic function equation is obtained:

(29) $$y=-5.36\cdot 10^{-11}x^{2}+4\cdot 10^{-7}x-0.01468$$

where x is time with unit of s and y is wavelength increment with unit of nm. This method can integrate the results of each experiment to a certain extent, and by applying different weights, the final equation is more strongly influenced by the experimental data with higher reliability. Similarly, the third curve from the five experiments can be fitted to obtain five linear equations:

(30) $$\begin{array}{l} y_{1}=3\cdot 10^{-6}x_{1}-0.0297\\ y_{2}=5\cdot 10^{-6}x_{2}-0.0408\\ y_{3}=4\cdot 10^{-6}x_{3}-0.0156\\ y_{4}=3\cdot 10^{-6}x_{4}-0.0263\\ y_{5}=4\cdot 10^{-6}x_{5}-0.0236 \end{array}$$

Note that the time when *x_i_* (i = 1, 2…5) is 0 in the above five equations refers to the time when the vortex pump is closed, and the dimension of *x* is seconds. Then the final functional equation is obtained by the weighted average method:

(31) $$y=3.8\cdot 10^{-7}x-0.0272$$

In summary, the piecewise function method combined with the weighted average method can be used to obtain the piecewise fitting equation for the two main change processes of measurement point Pt1-8FBGs-8 under leakage conditions during vacuum extraction.

Compared with the wavelength increment variations at the other measurement points, it is evident that the wavelength increments at points farther from the valve undergo a small increase and then remain essentially unchanged. Therefore, Equation (29) can be used to estimate the location of the leak. Under the same operating conditions, leakage is more likely to occur at locations where the wavelength increment profile closely follows Equation (29).

However, the measurement point Pt1-8FBGs-8, which is very close to the valve, experiences a larger strain due to stress concentration. This results in a relatively slower increase in the wavelength increment at Pt1-8FBGs-8 during this period, and consequently, the slope of the corresponding functional equation is smaller. Therefore, Equation (31) provides a useful reference for identifying the leakage location.

## 5. Conclusions

To develop an effective method for real-time monitoring of vacuum vessel leakage and to determine the actual leakage location of a vacuum tank, the stress–strain relationships of vacuum pressure vessel structures under different pressures were studied, and the stress concentration phenomenon at the edges of holes in thick-walled cylinder structures was also investigated. Leakage tests of the vacuum tank at different operating stages were conducted. Based on these studies, the following conclusions can be drawn:

(1) Through rigorous theoretical analysis of cylindrical pressure vessels under normal operating conditions, the stress–strain variation characteristics are accurately characterized. Furthermore, the stress distribution around circular holes is analyzed, and the manifestation and underlying mechanism of the stress concentration phenomenon are clarified to understand stress variations induced by leakage. This provides a solid theoretical foundation for an FBG sensor-based monitoring system, enabling accurate identification and localization of leakage locations based on abnormal stress changes caused by stress concentration.

(2) Analysis of multiple experimental datasets shows that the proposed monitoring system can characterize the real-time vacuum level curve by converting wavelength data measured by FBG sensors. This system can identify leakage and provide technical support for the safe operation and troubleshooting of the vacuum system.

(3) Based on the stress concentration phenomenon and the vacuum level curves obtained from the experiments, a preliminary judgment of the leakage location can be made. These observations suggest that FBG sensing technology combined with stress concentration theory can be effectively used to identify leakage faults. The experimental results verify the feasibility and effectiveness of this method for leakage detection in pressure vessels under practical engineering conditions.

Future work will focus on further improving the optical fiber-based monitoring system for vacuum pressure vessels by integrating refined theoretical models and algorithms, optimizing sensor deployment strategies, and enhancing the accuracy of real-time fault detection for vacuum vessels.

## Figures and Tables

**Table 1:** Geometrical and mechanical parameters of the vacuum vessel.

Contents	Tags	Value	Unit
Inner radius	*R*_1_	0.2	m
Outer radius	*R*_2_	0.205	m
External pressure	*P*_2_	0	Pa
Modulus of elasticity	*E*	200	GPa
Poisson ratio	*μ*	0.3	

## Data Availability

The original contributions presented in this study are included in the article. Further inquiries can be directed to the corresponding authors.
